# Construction and Development of a Cardiac Tissue-Specific and Hypoxia-Inducible Expression Vector

**DOI:** 10.15171/apb.2018.004

**Published:** 2018-03-18

**Authors:** Shahrooz Ghaderi, Neda Alidadiani, Jafar Soleimani Rad, Hamid Reza Heidari, Nafi Dilaver, Behzad Mansoori, Reza Rhabarghazi, Rezayat Parvizi, Vahid Khaze Shahgoli, Behzad Baradaran

**Affiliations:** ^1^Immunology Research Center, Tabriz University of Medical Sciences, Tabriz, Iran.; ^2^Department of Molecular Medicine, Faculty of Advanced Medical Sciences, Tabriz University of Medical Sciences, Tabriz, Iran.; ^3^Student research committee, Tabriz University of Medical Sciences, Tabriz, Iran.; ^4^Department of Anatomy, Faculty of Medicine, Tabriz University of Medical Sciences, Tabriz, Iran.; ^5^Department of Pharmaceutical Biotechnology, Faculty of Pharmacy, Tabriz University of Medical Sciences, Tabriz, Iran.; ^6^Swansea University Medical School, Swansea University, Swansea, UK.; ^7^Stem cell Research Center, Tabriz University of Medical Sciences, Tabriz, Iran.; ^8^Department of Applied Cell Sciences, Faculty of Advanced Medical Sciences, Tabriz University of Medical Sciences, Tabriz, Iran.; ^9^Department of Cardiothoracic Surgery, Faculty of Medicine, Tabriz University of Medical Sciences, Tabriz, Iran.

**Keywords:** Hypoxia, Hypoxia response element, Cis regulatory elements

## Abstract

***Purpose:*** Cardiovascular gene therapy is a sophisticated approach, thanks to the safety of vectors, stable transgene expression, delivery method, and different layers of the heart. To date, numerous expression vectors have been introduced in biotechnology and biopharmacy industries in relation to genetic manipulation. Despite the rapid growth of these modalities, they must be intelligently designed, addressing the cardiac-specific transgene expression and less side effects. Herein, we conducted a pilot project aiming to design a cardiac-specific hypoxia-inducible expression cassette.

***Methods:*** We explored a new approach to design an expression cassette containing cardiac specific enhancer, hypoxia response elements (HRE), cardiac specific promoter, internal ribosome entry site (IRES), and beta globin poly A sequence to elicit specific and inducible expression of the gene of interest. Enhanced green fluorescent protein (eGFP) was sub-cloned by BglII and NotI into the cassette. The specificity and inducible expression of the cassette was determined in both mouse myoblast C2C12 and mammary glandular tumor 4T1 as ‘twin’ cells. eGFP expression was evaluated by immunofluorescence microscope and flow cytometry at 520 nm emission peak.

***Results:*** Our data revealed that the designed expression cassette provided tissue specific and hypoxia inducible (O_2_<1%) transgene expression.

***Conclusion:*** It is suggested that cardiac-specific enhancer combined with cardiac-specific promoter are efficient for myoblast specific gene expression. As well, this is for the first time that HRE are derived from three well known hypoxia-regulated promoters. Therefore, there is no longer need to overlap PCR process for one repeated sequence just in one promoter.

## Introduction

Since identification and description of hypoxia-inducible factor 1 (HIF-1) by Wang *et al*.,^1^ more than 200,000 articles have been published in scientific literatures. HIF-1 is an inducible transcription factor, which binds to hypoxia response elements (HREs) or enhancer elements during hypoxia. HREs are located in the upstream of promoter region. HIF-1 regulates several genes, such as vascular endothelial growth factor (VEGF) and erythropoietin (EPO).^2^ hypoxia inducible factor-1 alpha (HlF-1) is comprised of a heterodimer basic helix-loop-helix (bHLH) transcriptional complex and is divided into distinct subsets, including HIF-1α and aryl hydrocarbon receptor nuclear translocator (ARNT), encoding a protein that is referred to beta (β) subunit. HIF-1 belongs to a conserved subfamily of PER-ARNT-SIM (PAS), which functions as oxygen sensors. The PAS domain is a subfamily of bHLH, which is a transcription factor. The HIF family consists of HIF-1α, -1β, -2α, -2β, -3α, -3β.^3,4^ In normoxia, HIF-1α is degraded by ubiquitin-mediated proteolysis activity, while being transcribed into mature RNA under hypoxic conditions, leading to an increase in oxygen delivery to the tissues. Therefore, HIF-1α has an essential role both in physiologic and pathologic conditions, including, but not limited to, myocardial ischemia, coronary artery disease, organ rejection and some cancers.^5,6^

Among all the above-mentioned circumstances, HIF-1α plays a great role in cardiovascular diseases. Lack of oxygen contributes to augmented hypoxia in coronary and cardiac tissue, subsequently leading to a cardiac stroke. Despite the emergence of novel surgical and medical approaches, cardiac infarction is set to become the leading cause of death up to the year 2020.^7^ Several reports have indicated that cardiac function, if not completely, then partially, can be ameliorated following gene or cell therapy in experimental models of infarction.^8,9^ However, ectopic gene expression has its disadvantages, such as hemangioma or uncontrolled vascular formation. It is, therefore, essential to regulate gene expression via the modulation of upstream elements of promoters like HREs. It should also be noted that hypoxia may subsequently exist in other tissues as a result of physiologic statement. Hence, tissue specific gene expression is essential to develop suitable vectors for gene therapy. This approach is also considered to be useful for sophisticated studies in the field of biomedical research such as cellular imaging and tracking,^10^ genome editing,^11^ stem cell engineering,^12^ and the study of signaling pathways.^13-15^

In this study, we investigated the cardiac specific promoter and cardiac specific enhancer combined with HREs consensus sequence.

## Materials and Methods

### Expression cassette designing and cloning 

**Figure 1:**
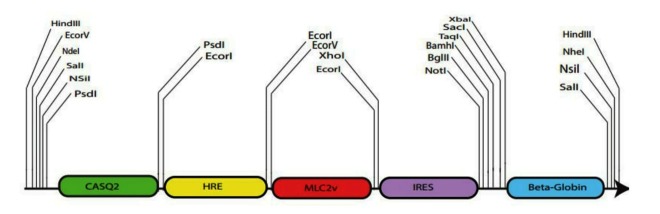
Schematic view of expression cassette. Expression cassette contains CASQ2 enhancer, HRE, MLC2v promoter, IRES, and β-globin poly A sequence.

**Figure 2:**
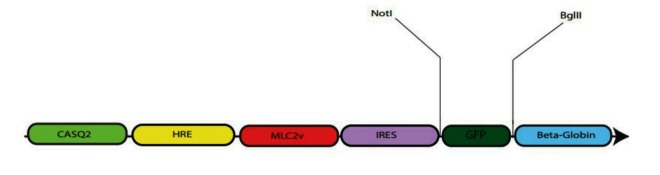
 Schematic view of eGFP cloning into the cassette. eGFP was cloned into the cassette by *NotI* and *BglII *restriction enzymes.

In order to construct the expression cassette, all the elements were synthesized by Generay Biotechnology Company (Shanghai, China) and sub-cloned into pGH cloning vector by *NdeI* and *NheI* restriction enzymes (Fermentas, Germany) (Figure 1). eGFP was sub-cloned into the cassette by *Bglll* and *Notl* (Figure 2). Briefly, 1 µg plasmid DNA was digested by *BglII and NotI* (Fermentas, Germany) and the relevant buffer was added up to the final volume of 20 µl in 37 °C for 1 h. Enzymatic reaction was inactivated by chloroform and the cloning procedure was confirmed by *HindIII* digestion.

### Bacterial strains and plasmid preparation 

Two strains of *Escherichia Coli* (E*. coli*) were used including DH5α and DH10β (Top10, Invitrogen, Thermo Fisher Scientific, USA). The plasmid was transformed into cells using the calcium chloride (CaCl_2_) method.^16^* E. coli* bearing the desired plasmid was cultured in lysogeny broth (LB) media for 16 hrs at 37 °C in a shaker incubator. Cells with a density of 3-4×10^9^ cells/ml with OD_600_=3 were harvested and DNA was extracted by plasmid DNA extraction kit (Qiagen, Midiprep Plasmid DNA Extraction Kit).

### Cell Culture 

The mouse myoblast cell line C2C12 (CRL-1772) and the mouse mammary gland cell line 4T1 (CRL-2539) was purchased from American Type Culture Collection (ATCC, Rockville, MD, USA). C2C12 cells were cultured in high glucose Dulbecco's Modified Eagle's Medium (DMEM) with 10% fetal bovine serum (FBS, Invitrogen, USA) and 0.584 g/L L-glutamine (Sigma-Aldrich, USA) in 37°C incubator and humidified 5% CO_2_ and 95% air.^17^ 4T1 cells, a 6-thioguanine resistant cell line,^18^ were cultured in RPMI-1640 (Sigma-Aldrich, USA) and 10% FBS (Invitrogen Gibco) in 37°C incubator and humidified atmosphere with 5% CO_2_ and 95% air. Cell lines were passaged after 80% confluence.

### The induction of hypoxic condition 

To create hypoxia, C2C12 and 4T1 cell lines were incubated for 90 min in a hypoxic condition, containing 94% N_2_, 4% CO_2_ and 1% O_2_, and hanks buffer. As a normoxia control “twin” cells were kept in a normoxic incubator.

### Plasmid Transient Transfection 

For achieving transient transfection, Gene Pulser XcellTM electroporation system (Bio-Rad, USA) was used. Following 60% confluence, media was removed and cells were harvested using 0.25% Trypsin-EDTA solution. Approximately 4-5 ×10^6^ cells were harvested by adding 3 ml complete media and then centrifuged for 5 min at 1000g and 4°C.^19^ Thereafter, cells were re-suspended in 400µl opti-MEM (buffer O).^20^ Then, 10µg of *supercoiled* DNA was overlaid to the cell suspension and mixed in wells. The cuvette was placed on ice for 5 min. Then, cells were transfected with the optimized exponential protocol (one shock for 18 seconds, at a voltage 120).

### Fluorescence microscopy 

Slides were visualized with a Zeiss Axioplan using 485 band pass filters set to view eGFP. All images were analyzed with AxioCam digital camera and Zeiss proprietary software (Axiovision Ver. 3.0.6.0). Images were manipulated in Adobe Photoshop 5.5.

### Flow cytometry 

eGFP expression was detected 48 hrs after transfection. Myocyte cells were harvested by trypsin/EDTA. Cells were centrifuged at 1000g for 10 min at 4°C. The cells were then washed three times with 500 µl of PBS. Fluorescence-activated cell sorting (FACS) caliber-micro flow cytometer (Becton Dickinson, NJ, USA) was used to analyze eGFP expression. GFP was excited by an argon laser and fluorescence’s at 485/520 nm band pass filter in the FL1 channel. All raw data were analyzed using FlowJo software version 7.6.1.

### Western Blotting 

Cells in the both hypoxia and normoxia groups were collected from the wells and their protein contents were detected using an extraction Kit (Santa Cruz, USA) following the manufacturer’s protocol. Total protein concentration was measured using a Nanodrop (Thermo-Scientific, USA). Samples were prepared for western blotting by adding loading buffer to each sample. Proteins were electrophoresed on 12% SDS-polyacrylamide gel and transferred to PVDF membranes. The membranes were blocked by incubating with 0.3 g bovine serum albumin in 10 ml of washing buffer at 4ºC overnight. Membranes were then washed three times with PBS for 10 min. Then, the membranes were incubated with anti-HIF-1α antibody (dilution: 1:500; Santa-Cruz) for 4 hrs at 4ºC. The membranes were then washed three times for 10 min each and incubated with the secondary antibody for 2 hrs. Roche ECL kit and semi-dry X-ray were used for imaging of immunoreactive protein bands.

## Results

### eGFP Cloning confirmation with HindIII digestion 

**Figure 3:**
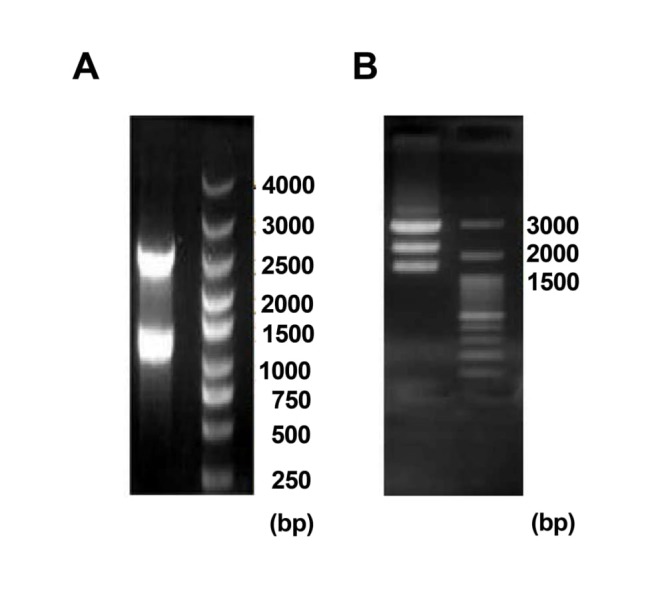
A; Confirmation of eGFP cloning into the cassette. Expression cassette was linearized by *HindIII* two sharp plasmid DNA band on 2500 and 1500 bps (Ladder 4000bps). B; Confirmation of eGFP cloning into the cassette by *HindIII* digestion three sharp plasmid DNA band on 3000, 2000 and 1500bps (Ladder 3000bps).

To confirm eGFP cloning, the expression cassette was digested by *HindIII* and positive colony was determined by three sharp plasmid DNA band, including 3000, 2000 and 1500 bps on gel electrophoresis. Meanwhile, empty expression cassette was digested into two bands on 2500 and 1500 bps (Figure 3A and B).

### Exploration of the CASQ2 enhancer and cardiac tissue specific promoter 

Calsequestrin 2 (CASQ2) as a cardiac specific enhancer plus myosin light chain-2 (MLC_2v_) cardiac specific promoter provided tissue specificity. Thanks to highly cardiac specific sequence expression, eGFP was detected in myoblast C2C12 cell line, but not in 4T1 ‘twin’ cell.

### Exploring of HRE 

**Figure 4:**
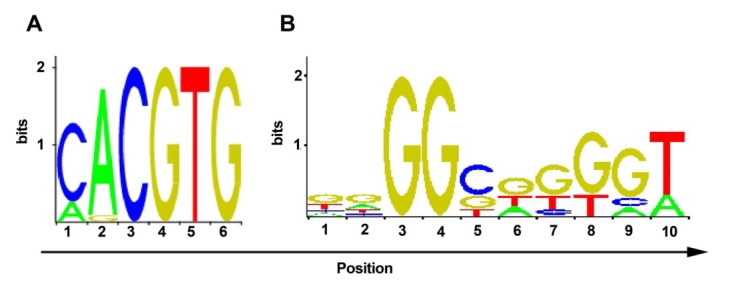
A; ARNT consensus sequence has been repeated 9 times in HRE. B; sp1 consensus sequence has been repeated 26 times in HRE

In this study, 35 putative sites, including 9 ARNT, HIF-1, and 26 sp1 from EPO, VEGF-A, and phosphoglycerate kinase 1 (PGK-1) promoters were driven (Figure 4A and B, Table 1). HRE sequence was analyzed for insulator boundary elements to prevent enhancer and promoter interaction (Table 2). The elements provided hypoxia inducible expression. eGFP was detected under hypoxia and was not expressed under normoxia condition.

### Hypoxia induction in C2C12 and 4T1 cell lines 

**Figure 5:**
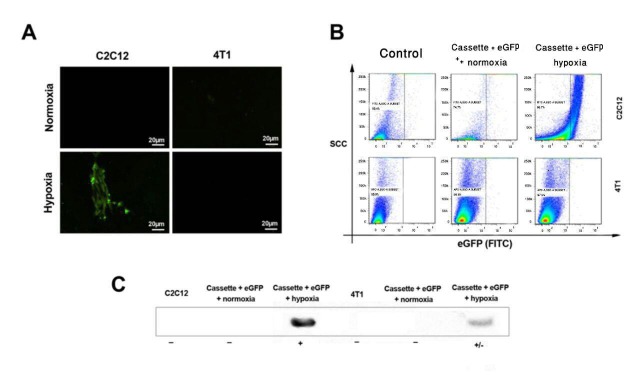
A; The results of immunofluorescence microscopy 48 hrs post transfection presented the specific expression of eGFP in the hypoxic condition at C2C12 myoblast cell line. eGFP was not detected in 4T1 cells in both hypoxic and normoxic condition. B; Flow cytometry results indicate the expression of eGFP under hypoxic condition in C2C12 cell line. C; Confirmation of hypoxia induction in both C2C12 and 4T1 cell lines by western blotting.

HIF-1α at transcriptional and translational levels was stabilized under hypoxic condition. Then, we detected HIF-1α protein in hypoxic condition. Notably, we could not detect any HIF-1α protein in normoxia (Figure 5C).

**Table 1:** 35 Putative sites that is existing on Hypoxia Responses Elements (HRE) including 9 ARNT and 26 sp1.

**The JASPAR database**							
**35 putative sites were predicted with these settings (80%) in sequence named 1**							
**Model ID**	**Model name**	**Score**	**Relative score**	**Start**	**End**	**Strand**	**predicted site sequence**
MA0079.2	SP1	6.736	0.800936630165687	27	36	1	CCCCGGCGAC
MA0079.3	SP1	1.668	0.802126030842755	38	48	1	CTTCCTGCTCC
MA0079.3	SP1	14.558	0.964296987823859	44	54	1	GCTCCGCCCCT
MA0079.2	SP1	9.501	0.870571327605649	45	54	1	CTCCGCCCCT
MA0079.1	SP1	6.122	0.800408014237765	46	55	­1	TAGGGGCGGA
MA0259.1	ARNT::HIF1A	8.788	0.928072894030867	81	88	1	CGGCGTGC
MA0259.1	ARNT::HIF1A	9.817	0.958770479367351	90	97	1	GGACGTGA
MA0259.1	ARNT::HIF1A	10.396	0.976043464702457	110	117	­1	AGACGTGC
MA0259.1	ARNT::HIF1A	9.025	0.935143183364926	145	152	1	ATACGTGG
MA0259.1	ARNT::HIF1A	10.321	0.973806031368894	174	181	1	CTACGTGC
MA0079.1	SP1	7.476	0.847637728498468	188	197	­1	CAGGCTGTGT
MA0079.2	SP1	6.736	0.800936630165687	212	221	1	CCCCGGCGAC
MA0079.3	SP1	1.668	0.802126030842755	223	233	1	CTTCCTGCTCC
MA0079.3	SP1	14.558	0.964296987823859	229	239	1	GCTCCGCCCCT
MA0079.2	SP1	9.501	0.870571327605649	230	239	1	CTCCGCCCCT
MA0079.1	SP1	6.122	0.800408014237765	231	240	­1	TAGGGGCGGA
MA0259.1	ARNT::HIF1A	6.391	0.856564524690194	266	273	1	GAGCGTGT
MA0259.1	ARNT::HIF1A	4.936	0.813158318019073	281	288	­1	CTACGGGC
MA0259.1	ARNT::HIF1A	8.854	0.930041835364402	306	313	­1	AAACGTGC
MA0259.1	ARNT::HIF1A	8.575	0.921718583363548	316	323	1	CCGCGTGC
MA0079.1	SP1	6.765	0.822836896268513	344	353	1	CAGGGGCGGT
MA0079.2	SP1	7.611	0.822972926823903	345	354	­1	CACCGCCCCT
MA0079.3	SP1	10.560	0.913997570755554	345	355	­1	GCACCGCCCCT
MA0079.1	SP1	7.289	0.841114865450646	346	355	1	GGGGCGGTGC
MA0079.3	SP1	3.163	0.820934842377801	350	360	­1	CTCCCGCACCG
MA0079.2	SP1	13.214	0.964080778453597	357	366	­1	CCCCGCCTCC
MA0079.3	SP1	13.420	0.949979644996513	357	367	­1	ACCCCGCCTCC
MA0079.1	SP1	11.046	0.972165113956893	358	367	1	GAGGCGGGGT
MA0079.3	SP1	3.129	0.820507083453258	362	372	­1	CCCACACCCCG
MA0079.2	SP1	8.685	0.850020906950673	364	373	­1	CCCCACACCC
MA0079.3	SP1	7.096	0.870416485031539	364	374	­1	GCCCCACACCC
MA0079.2	SP1	7.202	0.812672532157377	368	377	­1	CCCGCCCCAC
MA0079.2	SP1	8.757	0.851834179361406	369	378	­1	TCCCGCCCCA
MA0079.3	SP1	10.509	0.913355932368739	369	379	­1	ATCCCGCCCCA
MA0079.1	SP1	9.870	0.931144328158824	370	379	1	GGGGCGGGAT

**Table 2:** Insulator sequences have been located on HRE sequence.

**Motif** **PWM**	**Motif Sequence**	**Input** **Sequence** **Name**	**Motif** **Start** **Location**	**Motif** **Length**	**Motif** **Orientation**	**Score**
REN_20	CCTACGGGCACAGGGGACAC	1	87	20	+	-12.4488
MIT_LM2	GCGTCGCCGGGGGGCCCAC	1	153	19	+	-13.4637
MIT_LM2	GCGTCGCCGGGGGGCCCAC	1	338	19	+	-13.4637
MIT_LM7	GAGACAGCACGTAGGGCAAG	1	189	20	+	-7.41144
MIT_LM23	GAGACAGCACGTAGGGCAAG	1	189	20	+	-5.62159

### Expression cassette optimization 

After subsequent transfection (48 h and 72 h), HREs exhibited the most intense fluorescence 48 hrs after transient transfection only in the hypoxic condition. Both cell lines were incubated under normoxic and hypoxic conditions to determine the function of HREs, including HIF-1α and sp1 transcription factor binding site. The expression vector generated a sharp eGFP expression, which was detected by immunofluorescence microscope 48 hrs after transfection in the C2C12 cell line under hypoxic conditions. On the other hand, No significant eGFP was detected in 4T1 cells. eGFP was expressed in C2C12 cell line but not 4T1 under hypoxic (O_2_<1%) condition. Moreover, we cannot detect any significant eGFP signals in C2C12 under normoxia. Flow cytometry results showed that myoblast cells expressed eGFP in hypoxia. However, eGFP was not express in 4T1 cells in both hypoxia and normoxia. Overall, experiments demonstrated that our designed cassette was expressed and, hence, functioned appropriately (Figure 5A and B).

### eGFP measured 72h after transient transfection 

**Figure 6:**
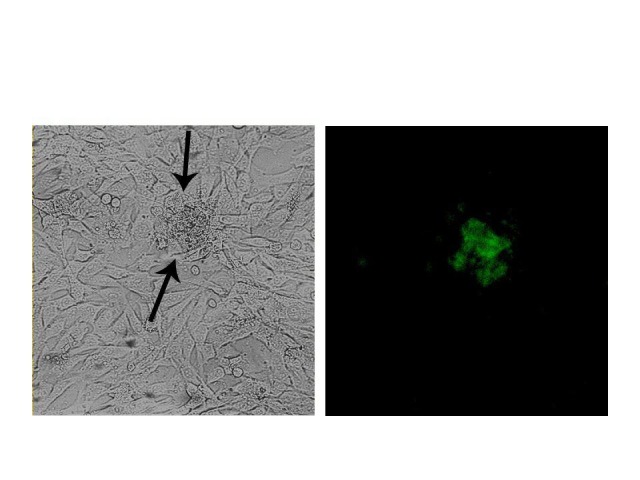
eGFP measurement 72 h after transfection. Arrow indicated the eGFP expression in C2C12.

After confirmation of hypoxia inducible and tissue specific expressions in expression cassette, the green fluorescence signals were analyzed 72 h and 96 h after transient transfection. eGFP signals were detected after 72h of transient transfection by the immune fluorescence microscopy at 520 band pass filter. However, we could not detect eGFP signals after 96 h after transfection (Figure 6).

## Discussion

The results obtained through the experiments support some of our hypotheses. The current study explored the application of bioinformatics to improve the efficiency of cardiac gene therapy. Based on *in silico* analysis, we chose a cardiac specific *cis*-regulatory conserved motif that belonged to *calsequestrin 2 (CASQ2)* gene. The corresponding gene is specifically expressed in the heart. Tissue-specific *cis*-regulatory elements and correlated transcription factors, including myogenic regulatory factors (MRFs) like myogenic differentiation 1 (MYOD1), myogenic factor 5 (MYF5), myogenin and myogenin regulator factor 4 (MRF4), myogenic factor 6 (MYF6) interact with other transcription factors, particularly T box protein 2 (TBX2) and NK2 homeobox 5 (Nkx2.5).,^21-24^ which play a pivotal role in the correct differentiation and progressive formation of cardiac muscle (Table 3). This summary helps us to understand the role of specific *cis*-regulatory elements in designing tissue-specific expression cassettes. Both CASQ2 and MLC_2v_ have a specific transcription factor binding site (TFBSs), which is efficient for cardiac-specific transgene expression.

Rincon *et al*.,^25^ previously showed that *CASQ2* had a strong specificity for cardiac muscle. They made a construct consisting of *CASQ2* and myosin heavy chain α (MHCα) as a cardiac specific promoter. In this study, we used MLC_2v_ because of its cardiac-specific regulatory elements (Table 4). For inducible expression under hypoxic conditions, we used HREs derived from phosphoglycerate kinase (PGK1), VEGF-165, and EPO promoter. However, it was shown that HRE was a conserved sequence itself, but the most noted HRE sequence was derived from the EPO promoter.^26^ To make copies of inducible elements obtained from one promoter, overlap PCR must be performed. For easier construct production of the desired elements, we used promoter and 5ʹUTRs of three different genes.^27,28^

HIF-1α belongs to a family of transcription factor with dimeric helices, containing basic amino acid residues that simplify DNA binding. bHLH proteins usually bind to a consensus sequence, well-known as HREs. Additionally, Sp1 is a zinc finger transcription factor that binds to GC-rich motifs of some promoters.^29,30^ Sp1 binding sites are located near the HRE regions on the promoter. More studies have shown that sp1 protein becomes overexpressed under hypoxic conditions.^31^ on the other side, hypoxic conditions have dual translation models known as cap-dependent and cap-independent (IRES) mechanisms. Notably, hypoxia has a strong restrictive effect on cap-dependent mRNA translation.^32^ However, cellular IRES has a significant role in adaptation to hypoxic stress but is not increased at translational level, possibly due to inhibition of protein synthesis in order to conserve energy. IRESs are natural translational enhancers and, hence, mediate internal initiation of translation when present between desired genes.

In clinical assays, bicistronic IRES-based expression vectors have fewer side effects than monocistronic based expression vectors. When we designed a vector for hypoxia-inducible expression, strong viral IRESs were used for efficient cap-independent gene expression. Fundamentally, the IRES-based expression vector is efficient for *in vitro* and *in vivo* gene expression under hypoxic conditions.^33^ Encephalomyocarditis virus (EMCV) is on-route into clinical studies and will hopefully be beneficial for patients. Our research, however, focused on *in vitro* studies. It is essential to study this cassette *in vivo* to evaluate all hypoxia-specific elements, such as enhancers (sp1, ARNT, and IRES) in a heterogenic population of myoblast and adult myocytes. However, we demonstrated that these elements were efficient for gene expression on hypoxic myoblasts in an *in vitro* model.

## Conclusion

In conclusion, combination of the cardiac/muscle specific cis-regulatory elements, *CASQ2*, and myosin light chain-2 (MLC_2v_) have a significant specificity for myocytes. Both *CASQ2* and MLC_2v_ have a specific TFBS, which is efficient for cardiac-specific transgene expression. Sp1 binding sites are located near the HRE regions on the promoter. Therefore, Sp1 and HIF-1α have binding sites to a far distance of HREs derived from three promoters. Therefore, there is no longer need to overlap PCR process for one repeated sequence just in one promoter. This expression cassette well-designed for cardiac specific hypoxia inducible gene expression.

**Table 3:** Cardiac specific transcription binding sites located on the CASQ2 enhancer.

**Model ID**	**Model name**	**Score**	**Relative score**	**Start**	**End**	**Strand**	**Predicted site sequence**
MA0037.2	GATA3	3.279	0.820594050245556	7	14	1	AGAAAAAC
MA0052.2	MEF2A	5.498	0.801872773397612	51	65	1	TACCTTACATAGCTC
MA0052.2	MEF2A	8.618	0.843873743315264	90	104	-1	TCCTAAAAATGGAGT
MA0083.2	SRF	8.230	0.817813133982383	90	107	-1	GCATCCTAAAAATGGAGT
MA0499.1	Myod1	5.403	0.859668968902211	105	117	1	TGCAGTTGTTTCA
MA0052.2	MEF2A	17.095	0.957989840107549	118	132	1	GGCTAAAAATAAATC
MA0063.1	Nkx2-5	5.189	0.833795488710155	136	142	-1	TTCATTG
MA0035.3	Gata1	2.402	0.800738582375976	151	161	-1	GTCGTATCTAA
MA0037.2	GATA3	6.588	0.872659931082488	153	160	1	AGATACGA
MA0482.1	Gata4	2.124	0.802327146192479	176	186	-1	CTGTATCAGCG
MA0499.1	Myod1	0.838	0.800984257597511	191	203	-1	TTCACCAGTCGGA
MA0482.1	Gata4	2.114	0.802183373083414	202	212	-1	TCTTCCCTCTT
MA0482.1	Gata4	6.727	0.868505908295031	223	233	-1	TCTTGTCTTTT
MA0036.2	GATA2	7.206	0.849841616420247	224	237	-1	ACATTCTTGTCTTT
MA0035.3	Gata1	6.525	0.857230665045272	224	234	-1	TTCTTGTCTTT
MA0037.2	GATA3	6.588	0.872659931082488	226	233	1	
MA0482.1	Gata4	6.776	0.869210396529448	244	254	-1	CCTTATTTCAT
MA0036.2	GATA2	5.069	0.820706175169384	245	258	-1	GCCTCCTTATTTCA
MA0035.3	Gata1	5.099	0.837692049986718	245	255	-1	TCCTTATTTCA
MA0037.2	GATA3	5.281	0.852094773556387	247	254	1	AAATAAGG
MA0482.1	Gata4	5.484	0.850634910838269	271	281	-1	CTTTCTCTTCT
MA0035.3	Gata1	2.802	0.806219259811756	272	282	-1	TCTTTCTCTTC
MA0037.2	GATA3	3.921	0.830695680797781	274	281	1	AGAGAAAG

**Table 4:** Specific transcription factor and their binding sites are Myosin Light Chain 2 (MLC2).

**Entrez_ID**	**Symbol**	**Alias**	**GO_P**	**GO_C**
4250	SCGB2A2	MGB1 UGB2	na	na
94234	FOXQ1	HFH1	transcription; regulation of transcription, DNA dependent; hair follicle morphogenesis	nucleus
6927	TCF1	HNF1 HNF1A LFB1 MODY3	Bone resorption; positive regulation of transcription from RNA polymerase II promoter	nucleus; transcription factor complex
6722	SRF	MCM1	Heart looping; transcription; signal transduction; multicellular organismal development; Heart development; positive regulation of transcription from RNA polymerase II promoter; muscle maintenance	nucleus
3170	FOXA2	HNF3B MGC19807 TCF3B	transcription; regulation of transcription, DNA dependent; lung development; epithelial cell differentiation; positive regulation of transcription from RNA polymerase II promoter; branching morphogenesis of a tube	nucleus
3169	FOXA1	HNF3A MGC33105 TCF3A	transcription; regulation of transcription, DNA dependent; lung development; epithelial cell differentiation; hormone metabolic process; glucose homeostasis; positive regulation of transcription from RNA polymerase II promoter; branching morphogenesis of a tube	nucleus
4763	NF1	DKFZp686J1293 NFNS VRNF WSS	cell cycle; Ras protein signal transduction; negative regulation of cell proliferation; regulation of glial cell differentiation; negative regulation of progression through cell cycle; regulation of small GTPase mediated signal transduction	intracellular; cytoplasm

## Acknowledgments

This work was a part of Ph.D. thesis and supported by the Research Council, Tabriz University of Medical Sciences. The authors thank the personnel of Immunology Research Center, Department of Pharmaceutical biotechnology for their kind guidance.

## Ethical Issues

Not applicable.

## Conflict of Interest

The authors declare “no” conflict of interest.

## Abbreviations

**HREs:** hypoxia response elements, **HIF-1α:** hypoxia inducible factor-1 alpha, **VEGF-A:** vascular endothelial growth factor-A, **PGK-1:** phosphoglycerate kinase 1, **MLC**_2v_**:** myosin light chain, **EPO:** erythropoietin, **ARNT:** aryl hydrocarbon receptor nuclear translocator, **eGFP:** enhanced green fluorescent protein, **CASQ2:** calsequestrin 2, **bHLH:** basic helix-loop-helix , **PAS:** PER-ARNT-SIM, **EMCV:** encephalomyocarditis virus, **UTR:** untranslated region, **IRES:** internal ribosome entry site, **MRFs:** myogenic regulatory factors, **MYOD1:** myogenic differentiation 1, **MYF5:** myogenic factor 5, **MYF6:** myogenic factor 6, **MHC-α:** myosin heavy chain- alpha, **TBX2:** T box protein 2, **NKX2-5:** NK2 homeobox 5.
